# Impact of Appropriate Antimicrobial Therapy for Patients with Severe Sepsis and Septic Shock – A Quality Improvement Study

**DOI:** 10.1371/journal.pone.0104475

**Published:** 2014-11-06

**Authors:** Paula K. O. Yokota, Alexandre R. Marra, Marines D. V. Martino, Elivane S. Victor, Marcelino S. Durão, Michael B. Edmond, Oscar F. P. dos Santos

**Affiliations:** 1 Division of Medical Practice, Hospital Israelita Albert Einstein, São Paulo, Brazil; 2 Laboratory of Microbiology, Hospital Israelita Albert Einstein, São Paulo, Brazil; 3 Statistics Department, Instituto Israelita de Ensino e Pesquisa (IIEP), Hospital Israelita Albert Einstein, São Paulo, Brazil; 4 Department of Internal Medicine, Virginia Commonwealth University School of Medicine, Richmond, Virginia, United States of America; Oxford University, Viet Nam

## Abstract

**Background:**

There is ample literature available on the association between both time to antibiotics and appropriateness of antibiotics and clinical outcomes from sepsis. In fact, the current state of debate surrounds the balance to be struck between prompt empirical therapy and care in the choice of appropriate antibiotics (both in terms of the susceptibility of infecting organism and minimizing resistance arising from use of broad-spectrum agents). The objective of this study is to determine sepsis bundle compliance and the appropriateness of antimicrobial therapy in patients with severe sepsis and septic shock and its impact on outcomes.

**Material:**

This study was conducted in the ICU of a tertiary care, private hospital in São Paulo, Brazil. A retrospective cohort study was conducted from July 2005 to December 2012 in patients with severe sepsis and septic shock.

**Results:**

A total of 1,279 patients were identified with severe sepsis and septic shock, of which 358 (32.1%) had bloodstream infection (BSI). The inpatient mortality rate was 29%. In evaluation of the sepsis bundle, over time there was a progressive increase in serum arterial lactate collection, obtaining blood cultures prior to antibiotic administration, administration of broad-spectrum antibiotics within 1 hour, and administration of appropriate antimicrobials, with statistically significant differences in the later years of the study. We also observed a significant decrease in mortality. In patients with bloodstream infection, after adjustment for other covariates the administration of appropriate antimicrobial therapy was associated with a decrease in mortality in patients with severe sepsis and septic shock (p = 0.023).

**Conclusions:**

The administration of appropriate antimicrobial therapy was independently associated with a decline in mortality in patients with severe sepsis and septic shock due to bloodstream infection. As protocol adherence increased over time, the crude mortality rate decreased, which reinforces the need to implement institutional guidelines and monitor appropriate antimicrobial therapy compliance.

## Background

Severe sepsis and septic shock are worrisome manifestations of systemic infection and the leading causes of hospitalization in intensive care units (ICUs), where an estimated 19 million cases occur worldwide each year, resulting in the death of one in four of these patients [1], [2]. According to the Ministry of Health of Brazil, severe sepsis and septic shock are responsible for an average of 41% of the total yearly in-hospital mortality for adults from 2008 through August 2013. Case mortality is 46% in the southeast region [3], which reinforced our desire to participate in the Surviving Sepsis Campaign beginning in 2004, as well as the “Stop Sepsis, Save Lives” project [4], [5].

According to the international guidelines, for the immediate treatment of severe sepsis or septic shock, antibiotic administration should occur within the first hour of recognition as it directly impacts in mortality [2]. Since 2006, we have utilized the sepsis bundle in our ICU [6], the treatment recommendations were organized in two bundles: a resuscitation bundle (6 tasks to begin immediately and to be accomplished within 6 hours) and a management bundle (4 tasks to be completed within 24 hours) [4]; however, there is no evaluation of the appropriateness of antimicrobial therapy after collecting blood cultures in sepsis bundle studies, which is one of the main measures to reduce mortality in critically ill patients suffering from infectious processes. When longer time frames to appropriate antibiotic therapy are observed there is an increased risk of death [7]–[9].

Thus, the objectives of the study were to determine i) the sepsis bundle compliance, ii) as well as the appropriateness of antimicrobial therapy for patients with severe sepsis and septic shock, as well as impact on outcomes.

## Methods

This study was conducted in the ICU of a tertiary care, private hospital in São Paulo, Brazil. This open model ICU is a 41-bed medical-surgical unit with approximately 10,500 patient-days yearly.

A retrospective cohort study was conducted from July 2005 to December 2012 in patients with severe sepsis and septic shock to analyze the impact of appropriate antimicrobial therapy. This study was a quality improvement project that was approved by the Institutional Review Board (IRB) of Hospital Israelita Albert Einstein. The requirements for informed consent were waived by our IRB in accordance of the Code of Federal Regulation and of the Privacy Rule. This project includes data from our previous sepsis bundle study from July 2005 to December 2009 [6] with the addition of observations for the period from January 2010 to December 2012 in order to evaluate whether sustained implementation of the sepsis bundle in our ICU could effectively reduce mortality in severe sepsis and septic shock patients.

Sepsis was defined as infection plus two or more of the following SIRS criteria: T>38°C or <36°C; heart rate >90/min; respiratory rate >20 breaths/min (or Paco2<32 mm Hg); or WBC count, >12,000 cells/µL or <4,000 cells/µL (or >10% band forms) [10]. Severe sepsis was defined as sepsis plus organ dysfunction, hypotension, or hypoperfusion abnormalities, including lactic acidosis, oliguria, or encephalopathy. Septic shock was defined as sepsis-induced hypotension (ie, systolic BP, <90 mm Hg or a drop of >40 mm Hg in the absence of other cause of hypotension) plus hypoperfusion abnormalities despite adequate fluid resuscitation [10] (Appendix S1) Infection was defined according to the definitions of the International Sepsis Forum [11] and adjudicated by the patient's physician.

The data collected included age, gender, admission date, the time when severe sepsis or septic shock was diagnosed, location before ICU admission, hospital and ICU length of stay, organ dysfunction at the time of diagnosis, APACHE II score on admission, and outcome status (death was defined as in-hospital mortality). As per the Surviving Sepsis Campaign, “time zero” was defined as the time of diagnosis of severe sepsis or septic shock.

Once a patient meets the bundle initiation criteria, the 6-hour bundle was initiated by collecting serum arterial lactate and obtaining blood cultures before antibiotic administration (Appendix S1). From the time of severe sepsis (time zero), broad-spectrum antibiotics are to be administered within 1 hour (since every hour of delay increases the risk of poorer outcomes). Hypotension and/or elevated lactate are treated with IV fluids; in the event of persistent hypotension despite fluid resuscitation (septic shock) and/or lactate >4 mmol/L (>36 mg/dL), maintaining adequate central venous pressure and central venous oxygen saturation are indicated. Patients who do not have septic shock and elevated lactate >4 mmol/L (>36 mg/dL) do not require measurement of central venous pressure and central venous oxygen saturation.

The first 24-hour management bundle includes optimization of glycemic control, respiratory inspiratory plateau pressure, and determination of the need for corticosteroids and drotrecogin alfa (activated) [4]. However, we did not perform analysis for the 24-hour sepsis bundle for patients with severe sepsis or septic shock because there have been changes in the recommendations (e.g., glycemic control, and use of activated protein C) [2], [6].

Our hospital has an electronic system for activating a team dedicated to diagnosing and treating severe sepsis and septic shock patients immediately. The ICU doctor and the nurse manager are simultaneously notified. The development of this sepsis team was part of implementing the sepsis bundle. Our hospital also has had a rapid response team since 2007 [6]. The rapid response team is alerted based on the following criteria: respiratory problems such as acute decrease in oxygen saturation <90% and change in respiratory rate to <8/minute or >28/minute; circulatory problems: decrease in systolic arterial pressure to <90 mmHg associated with symptoms; and change in heart rate to <40 bpm or >130 bpm; neurologic problems: decreasing consciousness levels or seizures; or a serious concern with the patient's overall condition (patient claims to be feeling unwell or has the sensation “something is not right”), and change in color, diaphoresis, or coolness of the extremities. Some of these described signs are the same as those noted in sepsis patients [6], [12].

### Antimicrobial therapy

Antimicrobial therapy was considered appropriate if the bacteria identified in blood culture was susceptible to at least one of the antibiotics administered within 24 hours after the collection of culture. If the isolated microorganism was not susceptible by in vitro testing to the antibiotic used, the therapy was considered inadequate [7].

The microbiology laboratory has an alert system to notify physicians of patients with positive blood cultures and their gram stain results.

### Microbiological methods

All samples were identified by manual or automated method and confirmed using the Vitek 2 (bio-Merieux Vitek, Inc., Hazelwood, MO) system. To determine the prevalence of antimicrobial resistance, the same organism with identical antimicrobial profiles from the same or different anatomic sites in the same patient were considered a single isolate. Antimicrobial susceptibility testing was performed by an automated method or by disk diffusion as described by the Clinical and Laboratory Standards Institute (CLSI) [13].

### Statistical analysis

Differences over time were evaluated by autoregressive time series models. The order of the models was determined according to the analysis of the partial autocorrelation function of residuals. The summary of the year was presented as means ± standard deviations or as absolute frequencies and percentages. Significance of changes was evaluated by trend effects. All tests of significance are two-tailed and alpha was set at 0.05.

To analyze the impact of inadequate antimicrobial therapy on mortality in the 358 patients with bloodstream infection, models were constructed by binary logistic regression in single and multiple approaches, only with data collected between 2010 and 2012. The variables with p≤0.10 in the simple analysis models were evaluated via multivariate analysis. The association of independent variables was expressed as odds ratios with 95% confidence intervals.

Analyses were performed with the Statistical Package for the Social Sciences 17.0 (Chicago, IL, USA).

## Results

The total cohort, shown in Figure S1, consisted of 1,279 consecutive patients, where 57.7% (N = 738) were male, mean age ± standard deviation (SD) 67±18 years, mean APACHE II score of 18±10, mean length of stay 33±71 days, mean time to administration of antibiotic was 2.5±3.6 hours, mean serum arterial lactate 27±24 mg/dL, and mean ± central venous oxygen saturation 76.2±11.4%. The total number of deaths was 371 (29.0%).

In analysis of sepsis bundle performance by year (table 1), there was a significant progressive increase in serum arterial lactate collection, administration of broad-spectrum antibiotic within 1 hour, and treatment with appropriate antimicrobial therapy. We also observed a significant decrease in crude mortality (figure S2).

**Table 1:** Demographic and clinical characteristics of severe sepsis and septic shock patients from July 2005 to December 2012.

Variables	Before protocol		After protocol													
	07/05 a 04/06		05/06 a 12/06		2007		2008		2009		2010		2011		2012	
	(n = 100)		(n = 73)		(n = 140)		(n = 134)		(n = 117)		(n = 197)		(n = 240)		(n = 279)	
	n	%	n	%	n	%	n	%	n	%	n	%	n	%	n	%
Septic shock	88	88.0	62	84.9	108	77.1	100	75.2	68	58.1	116	58.9	152	63.3	182	65.2
APACHE	25	±8	25	±9	24	±7	23	±6	21	±6	19	±7	21	±7	23	±7
Age	63	±20	66	±20	67	±19	67	±17	67	±18	69	±18	67	±18	66	±18
Serum arterial lactate	72	72.0	68	93.2	121	86.4	123	92.5	96	82.1	183	92.9	231	96.3	274	98.2
Blood culture prior to antibiotic administration	44	44.0	42	57.5	67	47.9	99	74.4	85	72.6	171	86.8	232	96.7	270	96.8
Positive blood culture	23	43.4%	27	34.6%	37	39.4%	41	37.3%	31	28.7%	52	30.4%	75	32.3%	72	26.7%
Broad-spectrum antibiotic within 1 h	58	58.0	46	63.0	105	75.0	103	77.4	73	62.4	105	53.3	137	57.1	228	81.7
Central venous oxygen saturation (ScvO_2_≥70%)	50	50.0	34	46.6	60	42.9	64	48.1	66	56.4	59	95.2	100	76.9	103	64.8
Appropriate antibiotic therapy	13	56.5%	19	70.4%	19	51.4%	32	78.0%	26	83.9%	40	76.9	52	69.3	59	81.9
Mortality Rate	54	54.0	30	41.1	55	39.3	55	41.4	19	16.2	61	31.0	46	19.2	49	17.6

-Analyses of antimicrobial appropriateness and value of central venous oxygen saturation were performed with the total of 199 and 358 patients, respectively.

-Extended data from the study published in Plos One 2011 (reference number 6).

In our cohort, 163 patients (12.7%) did not have blood culture collected. Of the 1,116 patients with blood cultures collected, 358 (32.1%) were positive. Comparing patients with and without positive blood cultures, cardiac dysfunction and a high APACHE II score were more prevalent in patients with positive blood cultures (p-value <0.001 and 0.005, respectively, table 2). Older patients and those with respiratory dysfunction were more likely to have negative blood cultures (p-value <0.001 and 0.05, respectively).

**Table 2:** Comparison between patients with and without positive blood cultures.

Variables	Blood culture		p
	Negative (N = 758)	Positive (N = 358)	
**Male - n (%)**	432 (57.%)	221 (61.7%)	0.134
**Organ dysfunction – n** (%)			
Liver	39 (5.1%)	27 (7.5%)	0.113
Cardiologic	492 (64.9%)	273 (76.3%)	<0.001
Renal	286 (37.7%)	138 (38.5%)	0.793
Hematologic	277 (36.5%)	134 (37.4%)	0.774
Respiratory	529 (69.8%)	210 (58.7%)	<0.001
Neurologic	260 (34.3%)	119 (33.2%)	0.727
Metabolic acidosis on admission	252 (33.2%)	133 (37.2%)	0.200
**Mortality rate – n (%)**	185 (24.4%)	101 (28.2%)	0.174
**Apache II - mean (±SD)**	16.8 (±10.1)	18.1 (±10.2)	0.005
**Age - mean (±SD)**	67.5 (±18.3)	64.2 (±17.9)	0.050

163 patients without blood culture collected during the study period.

For all patients with severe sepsis and septic shock, higher APACHE II scores were an independent predictor of mortality (Table 3). Patients with circulatory dysfunction, renal dysfunction, hematologic dysfunction, and respiratory dysfunction also had increased odds of death. Mortality was significantly lower in patients who had blood cultures obtained, and in those for whom serum arterial lactate was measured.

**Table 3:** Risk factors associated with death in all patients with severe sepsis or septic shock.

Variables	Survival (N = 908)	Death (N = 371)	Univariate analysis				Multivariate analysis			
			OR	CI 95%		P	OR	CI 95%		p
	N (%)	N (%)		Min	Max			Min	Max	
Age (years), mean ± SD	65.9±18	69.2±18	1.010	1.003	1.017	**0.004**	1.004	0.997	1.012	0.263
APACHE II, mean ± SD	16.2±9	21.8±10	1.060	1.046	1.074	**<0.001**	1.052	1.037	1.067	**<0.001**
Liver dysfunction	48 (5.2)	20 (5.3)	1.021	0.597	1.745	0.940				
Cardiac dysfunction	595 (97.8)	287 (77.3)	1.797	1.360	2.376	**<0.001**	1.439	1.048	1.976	**0.025**
Renal dysfunction	307 (33.8)	204 (54.9)	2.391	1.869	3.060	**<0.001**	1.792	1.371	2.341	**<0.001**
Hematologic dysfunction	307 (33.8)	155 (41.7)	1.405	1.096	1.800	**0.007**	1.684	1.270	2.234	**<0.001**
Respiratory dysfunction	576 (63.4)	255 (68.7)	1.267	0.979	1.640	**0.072**	1.693	1.265	2.266	**<0.001**
Neurologic dysfunction	283 (31.2)	142 (38.2)	1.369	1.064	1.762	**0.015**	1.286	0.976	1.694	0.073
Blood cultures prior to antibiotic administration	830 (91.4)	286 (77.0)	0.316	0.226	0.442	**<0.001**	0.380	0.264	0.546	**<0.001**
Broad-spectrum antibiotic within 1 hour	585 (64.4)	206 (55.5)	0.689	0.539	0.881	**0.003**	0.771	0.589	1.010	0.060
Serum arterial lactate	884 (97.4)	350 (94.3)	0.452	0.249	0.823	**0.009**	1.383	1.037	1.845	**0.027**
Central venous oxygen saturation	493 (54.3)	255 (68.7)	1.850	1.433	2.389	**<0.001**	0.392	0.203	0.757	**0.005**

OR  =  Odds Ratio; CI  =  Confidence Interval; APACHE: Acute Physiology and Chronic Health Evaluation II.

In the subset of patients with bloodstream infection, the APACHE II score were also remained an independent predictor of death (Table 4). Patients with renal and neurological dysfunction also had increased odds of death. After adjustment for other covariates, the administration of appropriate antimicrobial therapy was associated with a decreased risk of mortality (OR 0.54, p = 0.023) (Table 4). Higher APACHE II scores and the presence of polymicrobial infection were associated with inadequate antimicrobial therapy (p<0.001 and p = 0.016, respectively) (Table 5).

**Table 4:** Risk factors associated with death for patients with severe sepsis or septic shock and documented bloodstream infection.

Variables	Survival (N = 257)	Death (N = 101)	Univariate analysis				Multivariate analysis			
			OR	CI 95%		P	OR	CI 95%		p
	N (%)	N (%)		Min	Max			Min	Max	
Age (years), mean ± SD	64.5±17	63.2±19	0.996	0.983	1.009	0.521				
APACHE II, mean ± SD	16.9±10	21.0±11	1.041	1.017	1.066	**0.001**	1.029	1.004	1.055	**0.022**
Liver dysfunction	19 (7.4)	08 (7.9)	1.078	0.456	2.547	0.865				
Cardiac dysfunction	189 (73.5)	84 (83.2)	1.778	0.985	3.208	**0.056**	1.392	0.746	2.595	0.299
Renal dysfunction	81 (31.5)	57 (56.4)	2.815	1.754	4.518	**<0.001**	2.432	1.472	4.018	**0.001**
Hematologic dysfunction	89 (34.6)	45 (44.6)	1.517	0.949	2.425	**0.082**	1.583	0.953	2.628	0.076
Respiratory dysfunction	144 (56.0)	66 (65.3)	1.480	0.917	2.387	0.108				
Neurologic dysfunction	74 (28.8)	45 (44.6)	1.987	1.234	3.200	**0.005**	1.908	1.153	3.158	**0.012**
Broad-spectrum antibiotic within 1 hour	160 (62.3)	60 (59.4)	0.887	0.554	1.420	0.618				
Adequate antimicrobial therapy	197 (76.7)	63 (62.4)	0.505	0.308	0.829	**0.007**	0.536	0.314	0.916	**0.023**
Serum arterial lactate	252 (98.1)	97 (96.0)	0.481	0.127	1.829	0.283				
Central venous oxygen saturation	161 (62.6)	71 (70.3)	1.411	0.859	2.317	0.174				

OR  =  Odds Ratio; CI  =  Confidence Interval; APACHE  =  Acute Physiology and Chronic Health Evaluation II.

**Table 5:** Risk factors associated with inadequate antimicrobial therapy in patients with severe sepsis or septic shock patients who had documented bloodstream infection.

Variables	Adequate antimicrobial therapy		Univariate analysis				Multivariate analysis			
	No (N = 98)	Yes (N = 260)	OR	CI 95%		p	OR	CI 95%		p
	N (%)	N (%)		Min	Max			Min	Max	
Male	63 (28.5)	158 (71.5)	1.162	0.717	1.882	0.542				
Age (years), mean ± SD	65±20	64±17	1.005	0.991	1.018	0.495				
APACHE II, mean ± SD	21±11	17±10	1.044	1.02	1.069	**<0.001**	1.048	1.023	1.074	**<0.001**
Liver dysfunction	05 (18.5)	22 (81.5)	0.582	0.214	1.581	0.288				
Cardiac dysfunction	78 (28.6)	195 (71.4)	1.3	0.738	2.289	0.363				
Renal dysfunction	37 (26.8)	101 (73.2)	0.955	0.592	1.541	0.85				
Hematologic dysfunction	39 (29.1)	95 (70.9)	1.148	0.713	1.849	0.57				
Respiratory dysfunction	55 (26.2)	155 (73.8)	0.866	0.542	1.386	0.55				
Neurologic dysfunction	32 (26.9)	87 (73.1)	0.964	0.588	1.581	0.885				
Polymicrobial infection	12 (48.0)	13 (52.0)	2.651	1.165	6.032	**0.02**	2.954	1.227	7.115	**0.016**
ESKAPE pathogen causing bloodstream infection	44 (33.3)	88 (66.7)	1.593	0.992	2.558	**0.054**	1.436	0.872	2.366	0.155

OR  =  Odds Ratio; CI  =  Confidance Interval; APACHE  =  Acute Physiology and Chronic Health Evaluation II.

**ESKAPE  =** ***Enterococcus, Staphylococcus aureus, Klebsiella pneumoniae, Pseudomonas aeruginosa, Enterobacter spp.***

Among the 358 patients with positive blood cultures, 333 (93.0%) had monobacterial infections (table 6). The most common pathogens were *Escherichia coli* (n = 101, 28.2%), *Streptococcus pneumoniae* (n = 31, 8.7%). Bacteria belonging to the ESKAPE group were represented as follows: *Staphylococcus aureus* (n = 24, 6.7%), *Klebsiella pneumoniae* (n = 20, 5.6%), *Pseudomonas aeruginosa* (n = 18, 5.0%), *Enterobacter* spp (n = 10, 2.8%), *Enterococcus faecium* (n = 2, 0.6%), *Acinetobacter baumannii* (n = 4, 1.1%). Considering fungal infections, 5 patients (1.5%) had *Candida* bloodstream infection; of these, 2 patients had *Candida albicans*, 2 patients had *Candida parapsilosis*, and one patient *Candida krusei*. Among the monomicrobial infection cases 74.2% (n = 247) received adequate antimicrobial therapy. One reason for inadequate therapy was fungal bloodstream infection (83.3%, n = 5). There were few cases of polymicrobial bloodstream infections (N = 25, 7.0%). More than a half of these cases received adequate antimicrobial therapy (n = 13, 52.0%). Highly resistant organisms not covered by usual empiric therapy as represented by the ESKAPE pathogen group could be expected to be associated with inadequate therapy; however, we were not able to demonstrate this by the multivariate analysis (table 5).

**Table 6:** The most prevalent microorganisms from monomicrobial bloodstream infections and clinical outcome stratified by adequacy of antimicrobial therapy during the study period.

Microorganisms		All organisms					Resistant organisms				
		N (%)	Death		Survival		N (%)	Death		Survival	
			Adequate ATB	Inadequate ATB	Adequate ATB	Inadequate ATB		Adequate ATB	Inadequate ATB	Adequate ATB	Inadequate ATB
			N (%)	N (%)	N (%)	N (%)		N (%)	N (%)	N (%)	N (%)
Fungi (N = 5, 1.5%)	*Candida albicans*	02	-	01	01	-	no	-	-	-	-
		(40.0)		(50.0)	(50.0)						
	*Candida krusei*	01	-	-	-	01	01*	-	-	-	01
		(20.0)				(100.0)	(100.0)				(100.0)
	*Candida parapsilosis*	02	-	02	-	-	no	-	-	-	-
		(40.0)		(100.0)							
Gram negative (N = 190; 57.1%)	*Escherichia coli*	101	13	05	77	06	07**	01	04	02	-
		(53.1)	(12.8)	(4.9)	(76.2)	(6.1)	(6.9)	(14.2)	(57.3)	(28.5)	
	*Pseudomonas aeruginosa*	18	07	04	06	01	08#	02	04	02	-
		(10.0)	(38.9)	(22.2)	(33.3)	(5.6)	(44.4)	(25.0)	(50.0)	(25.0)	
	*Acinetobacter* spp	8	02	01	04	01	01#	-	01	-	-
		(4.2)	(25.0)	(12.5)	(50.0)	(12.5)	(12.5)		(100.0)		
	*Klebsiella* spp	24	03	04	13	04	10**	01	03	03	03
		(12.6)	(12.5)	(16.6)	(54.3)	(16.6)	(41.2)	(10.0)	(30.0)	(30.0)	(30.0)
Gram positive (N = 138; 41.4%)	*Staphylococcus aureus*	24	07	04	02	11	11##	06	04	01	-
		(17.3)	(29.2)	(16.6)	(8.4)	(45.8)	(45.8)	(54.5)	(36.3)	(9.2)	
	*Enterococcus* spp	11	04	00	04	03	01###	-	-	01	-
		(7.9)	(36.3)	(0.0)	(36.3)	(27.4)	(9.2)			(100.0)	
	Coagulase-negative staphylococci	44	13	08	16	07	35##	11	08	11	05
		(26.1)	(29.5)	(18.2)	(36.4)	(15.9)	(79.5)	31.4)	(22.9)	(31.4)	(14.3)

*resistant to fluconazole.

**resistant to 3rd and 4th generation cephalosporins.

# resistant to carbapenems.

## resistant to methicillin.

### resistant to vancomycin.

## Discussion

With improvement of compliance to the sepsis bundle over time, we demonstrate an association with reduced mortality. Not surprisingly, risk factors for death include the APACHE II score, as well as the presence of other disorders that contribute to severe worsening of the patient's general condition [1].

In addition to implementing the sepsis protocol at our institution (May 2006), practical bedside contact with evidence-based medicine and attendance at several scientific meetings by the multidisciplinary team also contributed in improving the care of these patients. Over the period of analysis, changes in treatment, such as the utilization of Activated protein C followed by its suspension reflect the need for continuous learning and improving based on the state of the science [6].

Another important fact, demonstrated by logistic regression, is that despite the implementation of the measures incorporated in the sepsis bundle, such as collecting serum arterial lactate and timely administration of antibiotics, it was antimicrobial appropriateness that contributed to decreased mortality in severe sepsis and septic shock patients. Over the past two years, our mortality rate in severe sepsis and septic shock patients was below 20% as compared to 55% prior to the implementation of the sepsis bundle [6].

Over time, after implementation of the bundle, we observed improved compliance with the protocol (Figure S2); in 2012 we had 54% compliance with the sepsis bundle, and a 17% mortality rate. The correct antibiotics, administered at the right time, contribute to an effective recovery of the patient. It also acts as a measurable and actionable safety metric, which reduces mortality by decreasing the time of exposure to the organism and its toxins. This has been the target of study of several groups around the world [14]–[17]. However, the indiscriminate use of antibiotics may lead, for example, to the development of antimicrobial resistance and an increase in *Clostridium difficile* infections. In addition, some antimicrobials have side effects that require caution and rational prescribing [7]–[9].

While empiric antibiotic therapy is important [17], targeted therapy based on culture results should also be a goal. However, sepsis bundle evaluation studies have not examined the appropriateness of the antimicrobial therapy administered [18].

Because identification of pathogens in cultures is time intensive, one of the ways that our institution found to speed up the process of communicating blood cultures results was for laboratory staff to call the physicians responsible for the patient.

Of the ESKAPE pathogens [19], [20], which have paramount importance due to the their pathogenesis, transmission mode and antimicrobial resistance, the most common was *E.coli*. However, the incidence of *S. aureus* (MRSA) and *E. faecium* (VRE) cases was low (Table 6).

Regarding the appropriateness of antibiotic selection for the cases with positive blood culture, our data show a fair rate of treatment adequacy, but in relation to fungal infections of the five cases identified 60.0% (3/5) died and none were receiving adequate therapy (Table 6). In the APACHE II study [21], calculations were done only on the day of admission. One explanation to justify why patients with higher APACHE II were associated with inadequate antimicrobial therapy was in the first 24 hours there was an underestimation of the severity of these patients and the appropriate antimicrobial therapy. If we had calculated the prognostic score on our patients at the time of severe sepsis and septic shock diagnosis, the APACHE score would likely be very different. Recently, in a similar study evaluating etiology, antimicrobial therapy and outcome of patients with severe sepsis, quick identification of the source of infection, proper sampling for microbiological analyses, and the rapid administration of adequate antimicrobial therapy were shown to be crucial management points [22].

### Study Limitations

A limitation of our study is that it was performed at a single private medical facility so it may not be generalizable to other hospitals (e.g., public facilities). In addition, data was retrospectively collected from medical records; however because of the sepsis bundle protocol these septic patients were followed prospectively. Lastly, we did not include microbiologic data from sites other than blood cultures with regards to assessing appropriateness of antimicrobial therapy. However, cultures from nonsterile sites (e.g., respiratory tract) are more difficult to interpret since in many cases the organisms are colonizing rather than causing true infection.

### Implications for policy and practice

Other improvements that were taking place may have impacted our study findings. Since 2007 our hospital has been engaged in zero tolerance for healthcare associated infections. We have observed a significant reduction in ventilator-associated pneumonia and in central venous associated bloodstream infections [23], [24]. We also implemented other ICU best practices during the study period, including a glycemic control protocol [25]. We adopted intermediate glucose control, because we believe that tight glucose control is difficult to accomplish in routine intensive care unit settings and is associated with a significant increase in the incidence of hypoglycaemia [25], [26]. However, it is interesting to note that the best practices for the care of patients with severe sepsis and septic shock were improving over time in our ICU. This affirms our belief that the sepsis bundle needs to be considered as the intervention decreasing mortality in septic shock and severe sepsis because of the better care and the prompt recognition of these patients in hospital [6].

## Conclusions

Appropriate antimicrobial therapy and implementation of the sepsis bundle in patients with severe sepsis and septic shock due to bloodstream infection was associated with a 46% reduction in mortality. Assessment of appropriate antimicrobials should be incorporated into the Surviving Sepsis Campaign as a quality metric [18].

## Supporting Information

Figure S1 Flow-diagram - Cohort selection of total patients, septic shock patients and bloodstream infection.
(TIF)

Figure S2 Proportion of patients with severe sepsis and septic shock who died and sepsis bundle compliance.
(TIF)

Appendix 1 Sepsis definitions.
(DOCX)
