# Supplementary figures and images for: Impact of Appropriate Antimicrobial Therapy for Patients with Severe Sepsis and Septic Shock – A Quality Improvement Study
Source: PLoS One. 2014 Nov 6;9(11):e104475. doi: 10.1371/journal.pone.0104475 (PMC4222820; doi:10.1371/journal.pone.0104475)

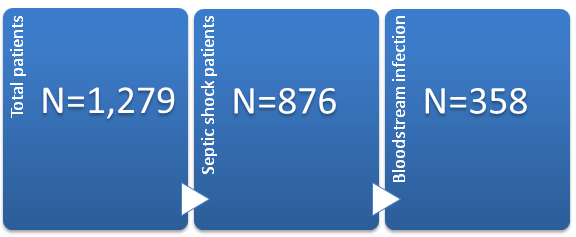

Supplement: Figure S1 — Flow-diagram - Cohort selection of total patients, septic shock patients and bloodstream infection. (TIF) [file pone.0104475.s001.tif]

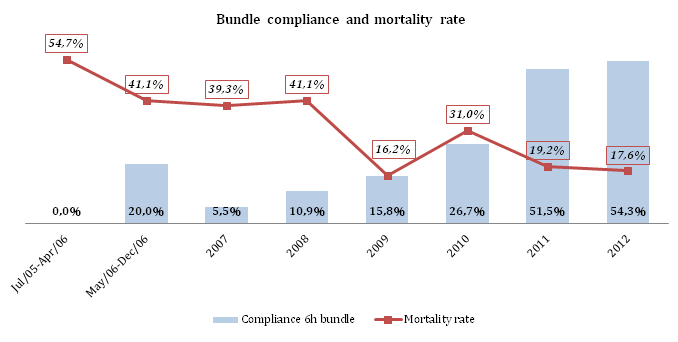

Supplement: Figure S2 — Proportion of patients with severe sepsis and septic shock who died and sepsis bundle compliance. (TIF) [file pone.0104475.s002.tif]
