# Supplementary material for: Impact of Appropriate Antimicrobial Therapy for Patients with Severe Sepsis and Septic Shock – A Quality Improvement Study
Source: PLoS One. 2014 Nov 6;9(11):e104475. doi: 10.1371/journal.pone.0104475 (PMC4222820; doi:10.1371/journal.pone.0104475)

**Appendix 1**. Sepsis definitions.

| **Sepsis** was defined as infection plus two or more of the following SIRS criteria: T >38°C or <36°C; heart rate >90/min; respiratory rate >20 breaths/min (or Paco2 <32 mm Hg); or WBC count, >12,000 cells/μL or <4,000 cells/μL (or >10% band forms). |
| --- |
| **Severe sepsis** was defined as sepsis plus organ dysfunction, hypotension, or hypoperfusion abnormalities, including lactic acidosis, oliguria, or encephalopathy. |
| **Septic shock** was defined as sepsis-induced hypotension (ie, systolic BP, <90 mm Hg or a drop of >40 mm Hg in the absence of other cause of hypotension) plus hypoperfusion abnormalities despite adequate fluid resuscitation.  **Sepsis bundle**: A) The 6-hour bundle includes: colllecting serum arterial lactate, obtaining blood cultures before antibiotic administration, broad-spectrum antibiotics within 1 hour. B) The first 24-hour management bundle includes optimization of glycemic control, respiratory inspiratory plateau pressure, and determination of the need for corticosteroids and drotrecogin alfa (activated) – we did not perform analysis for the first 24-hour because there have been changes in the sepsis recommendations [2]. |


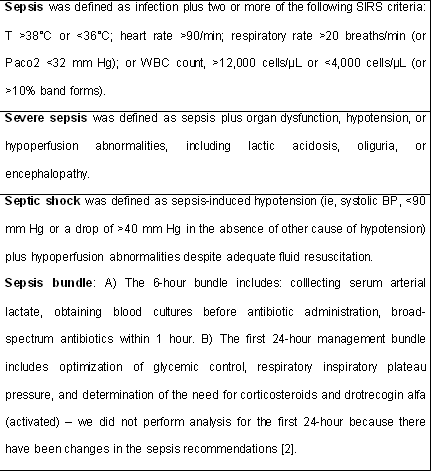

Supplement: Appendix 1 — Sepsis definitions. (DOCX) [file pone.0104475.s003.docx]
